# Measuring and Preventing COVID-19 Using the SIR Model and Machine Learning in Smart Health Care

**DOI:** 10.1155/2020/8857346

**Published:** 2020-11-02

**Authors:** Saad Awadh Alanazi, M. M. Kamruzzaman, Madallah Alruwaili, Nasser Alshammari, Salman Ali Alqahtani, Ali Karime

**Affiliations:** ^1^Department of Computer Science, College of Computer and Information Sciences, Jouf University, Sakakah, Saudi Arabia; ^2^Department of Computer Engineering and Networks, College of Computer and Information Sciences, Jouf University, Sakakah, Saudi Arabia; ^3^Department of Computer Engineering, College of Computer and Information Sciences, King Saud University, P.O. Box: 51178, Riyadh 11543, Saudi Arabia; ^4^Department of Electrical and Computer Engineering, Royal Military College of Canada, Kingston, Canada

## Abstract

COVID-19 presents an urgent global challenge because of its contagious nature, frequently changing characteristics, and the lack of a vaccine or effective medicines. A model for measuring and preventing the continued spread of COVID-19 is urgently required to provide smart health care services. This requires using advanced intelligent computing such as artificial intelligence, machine learning, deep learning, cognitive computing, cloud computing, fog computing, and edge computing. This paper proposes a model for predicting COVID-19 using the SIR and machine learning for smart health care and the well-being of the citizens of KSA. Knowing the number of susceptible, infected, and recovered cases each day is critical for mathematical modeling to be able to identify the behavioral effects of the pandemic. It forecasts the situation for the upcoming 700 days. The proposed system predicts whether COVID-19 will spread in the population or die out in the long run. Mathematical analysis and simulation results are presented here as a means to forecast the progress of the outbreak and its possible end for three types of scenarios: “no actions,” “lockdown,” and “new medicines.” The effect of interventions like lockdown and new medicines is compared with the “no actions” scenario. The lockdown case delays the peak point by decreasing the infection and affects the area equality rule of the infected curves. On the other side, new medicines have a significant impact on infected curve by decreasing the number of infected people about time. Available forecast data on COVID-19 using simulations predict that the highest level of cases might occur between 15 and 30 November 2020. Simulation data suggest that the virus might be fully under control only after June 2021. The reproductive rate shows that measures such as government lockdowns and isolation of individuals are not enough to stop the pandemic. This study recommends that authorities should, as soon as possible, apply a strict long-term containment strategy to reduce the epidemic size successfully.

## 1. Introduction

The rapid growth of COVID-19 has forced scientists to develop urgent countermeasures to halt the outbreak. Scientists have proposed and implemented various technologies to reduce the negative consequences of the pandemic and to accelerate the recovery phase [1]. These technologies include artificial intelligence (AI), machine learning, deep learning, cloud-based collaboration tools, fog computing, the Internet of Things (IoT), cognitive computing, and wireless communication. There is great potential for these technologies to bring about a revolution in the healthcare industry [2–4].

AI, and in particular machine learning algorithms, has increasingly become an integral part of smart healthcare. These technologies are increasingly referred to as the brain of smart healthcare services [5, 6]. Deep learning, a subset of machine learning in AI, has networks capable of learning—unsupervised—from unstructured or unlabeled data, and has been intensively used in many applications including COVID-19 [7–10]. In deep learning, convolutional neural networks (CNN) are a class of deep neural networks, most commonly applied in the field of computer vision [11]. This method has been applied to many tasks, including super resolution, image classification, semantic segmentation, multimedia systems, healthcare, and emotion recognition. [12–16].

On the other hand, cloud computing can provide the digital infrastructure needed for smart health care. That is, smart human care services cloud could provide data storage and data processing for all activities [17–20]. Fog computing is the latest computing paradigm and employs user or close user devices, also called the network edge or edge users, to perform data processing tasks. This network architecture enhances the flexibility of cloud computing as compared to more ubiquitous networks [21, 22]. The Internet of Things is a set of interrelated computing devices with unique identifiers and enables the network transfer of data without requiring human-to-human or human-to-computer interaction. It includes everything from desktops, laptops, and smartphones, to coffee makers, washing machines, and wearable devices [23–28].

Smart healthcare services require formal processes for measuring, preventing, and managing the spread of COVID-19. The technologies mentioned can be used to reduce this pandemic's negative consequences and accelerate the recovery phase. Various models have been used to study how the virus spreads across populations: susceptible-infected (SI), susceptible-infected-recovered (SIR), susceptible-infected-susceptible (SIS), and the susceptible-infected-recovered-susceptible (SIRS) models [29]. These models offer two possible outcomes. The first possibility is that the disease might, if new infections are not controlled, end up being an epidemic. The second outcome is that the virus dies off if the necessary measures are taken to protect susceptible individuals from infection. COVID-19 follows a similar pattern to that of other infectious diseases and contact tracing is needed to help reduce new infections. The similarities between COVID-19 and other infectious diseases in terms of contact infections make it possible to predict its outcomes. Two models are potentially useful in managing the disease. These are the SIR and SIR-F models, in which F stands for “fatal with the confirmation.” The idea is to ensure that people who recover are not again susceptible to the virus. The challenge, however, is that human behavior does not follow one set of specific rules but rather tends to change from one community to another and from one person to another. It is thus difficult to accurately use these models to determine the future outcomes of the disease, but the models can help in developing real-life approaches to the virus.

Machine learning (ML) algorithms for time-series forecasting are statistics and computer science, where calculations are derived from the data. Scikit-Learn is a machine-learning library for Python. It is also a community-driven project with powerful regression tools for fitting curves to the table of suspected and recovered cases. ML can assist in establishing overarching information on the pandemic and in forecasting the advancement of infections. The various epidemic models can be evaluated with COVID-19 data using the model parameter estimation package CovsirPhy. SIR-F is a customized SIR-derived ODE model [30].

A discrete-time stochastic compartment model was used in [31] to study the dynamics of the COVID-19 epidemic. This model forecasts the spread of the disease in the next period based on parameter estimates and numerical simulation. In [32], mathematical and numerical analyses were carried out using a time-based SIR model for COVID-19 with asymptomatic individuals. In another study, AI predictions and a modified SEIR (Susceptible-Exposed-Infectious-Recovered-Susceptible) model were used to study the COVID-19 epidemic trends in China. This is helpful in understanding the public health interventions applied [33]. The SEIR model was effective in predicting the COVID-19 trends and the infection rates. The AI-based model was developed based on a SARS dataset that suggests that there is hope for managing the pandemic. To validate the data, an advanced SIR prediction model was applied to the epidemic data from Italy and compared against the results from China [34].Extended SIR forecast of the COVID-19 epidemic trend in Italy is studied in [35], whereas introduction of population migration with an effective intervention approach for COVID-19 is introduced in [36]. The SIR model incorporating time-based parameters and AI algorithms was also used to study the spread of COVID-19 in South Korea [37]. However, we have not found related research for smart healthcare.

This study presents a more accurate prediction model for smart healthcare services using a machine learning approach with the SIR model. The proposed model works with a stochastic model for analyzing the COVID-19 pandemic, and we then investigate time-series forecasting of COVID-19 for the next 700 days. We began with an exploratory data analysis of COVID-19 datasets belonging to John Hopkins University. These datasets include all countries although for some there is no detailed information about the number of patients hospitalized, or of interventions of the government to lockdown institutions, schools, and markets. In this study, we considered modeling data for the Kingdom of Saudi Arabia (KSA) only and including other countries as would be the case in a global model so as to evaluate the effectiveness of the intervention in curbing the spread of COVID-19 [38].

## 2. Data Analysis

Most countries have a COVID-19 dataset. Mortality, recovery, and infection rates are essential for the SIR model. The datasets present social behavior data but not for all countries. The population data are the starting point of our analysis.

### 2.1. Population

In the global model, we used population numbers for all countries. For each of the simple models, we made use of a general population pyramid across different age groups employing the real population pyramid of Table 1. In Table 1, people are categorized into 5 age groups, separated by the total number of males and females.

Individuals in the various age groups spend their time in different places: children are at school, people of working age are mostly in offices or other workspaces, and retired persons are at home. The number of hours that the people are awake on a daily basis is given in Table 2.

### 2.2. Johns Hopkins Dataset

The Johns Hopkins University (JHU) datasets are arguably the most popular COVID-19 data sources available. Data for about 181 countries are updated regularly. The number of confirmed, infected, fatal, and recovered cases for KSA as of 12 June 2020 and obtained from the JHU is shown in Table 3 [38].

**Figure 1:**
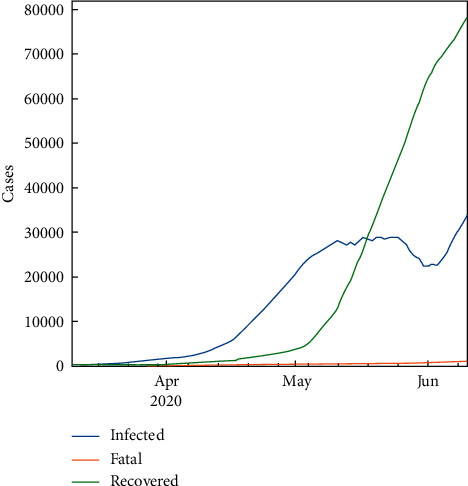
Number of cases.

**Figure 2:**
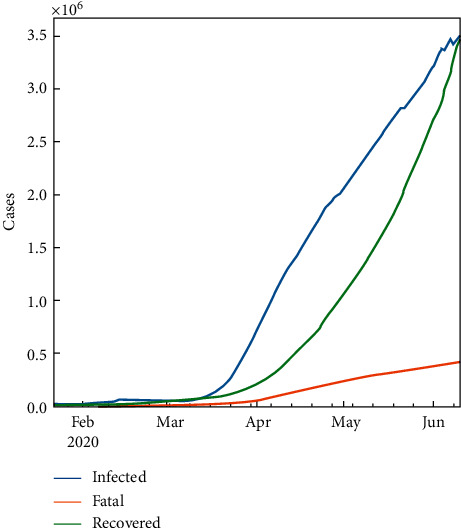
Total number of cases in the world.

The exploratory data analysis (EDA) of COVID-19 for KSA is summarized in Table 4 and Figure 1. For purposes of comparison, the total number of cases globally can be seen in Figure 2.

### 2.3. Evaluation of the SIR Model

In this section, we construct a mathematical model derived from an SIR model. An SIR model is a basic statistical tool for analyzing infectious disease outbreaks. We will evaluate this in the upcoming sections. Unmonitored symptomatic cases that are a source of infection in the population are not considered. There is also a chance of being infected through touching objects that are infected. There are numerous random factors relevant to disease transmission. Among the many possibilities, we propose the stochastic SIR model detailed below. At the end of the initial modeling, we produced the SIR-F model, a custom ODE SIR-derived model. A parameter estimate for SIR-F will be applied to subsets of time-series data in each country to determine the impact of interventions.

### 2.4. Basic SIR Model

In this basic model, the total population is divided into three subsets: susceptible, infected, and recovered. There are transitions between these parts. Susceptible persons are derived by subtracting persons who are confirmed to be virus carriers via testing in hospitals from the total population. Moving from the susceptible to the infected cases, the contact rate, *β*, determines the disease velocity in the population. In detail, the transition from *S*-state to *I*-state is not deterministic, but is always stochastic. Hence, *β* includes multiplication of rate, probability, and population number. The basic model can be defined as

(1) $$\begin{array}{c} S\overset{\beta I}{⟶}I\overset{\gamma }{⟶}R, \end{array}$$

where *S*: susceptible = all − confirmed; *I*: infected =confirmed − recovered − deaths; *R*: recovered or fatal =recovered + deaths; *β*: effective contact rate (1/min); and *γ*: recovery (+mortality) rate (1/min).

The number of infected persons decreases with recoveries and deaths. Recovered individuals can also no longer change to the susceptible state.

Ordinary Differential Equations (ODE) of the basic SIR model are given below [39]:

(2) $$\begin{array}{c} \frac{\text{d}S}{\text{d}T}=-N^{-1},\\ \frac{\text{d}I}{\text{d}T}=N^{-1}\beta SI-\gamma I,\\ \frac{\text{d}R}{\text{d}T}=\gamma I, \end{array}$$

where *N*  = *S* + *I* + *R* is the total population, *T* is the elapsed time from the start date. ODE is used for the simulation to compute the variables after a short period of time. At any point in time, the ODE shows the rate of change for every variable. We can forecast the future by bringing together little changes over time. Thus, the SIR model is used for three main differential equations.

To implement the ODE functions mentioned, we simplify them as follows:

(3) $$\begin{array}{c} \frac{\text{d}x}{\text{d}t}=-\rho xy,\\ \frac{\text{d}z}{\text{d}t}=\sigma y. \end{array}$$

(4) $$\begin{array}{c} R_{0}=\rho \sigma^{-1}=\beta \gamma^{-1}, \end{array}$$

where *R*_0_ is the new term signifying the reproductive rate (contact rate), which means that one infection will cause several new infections. The infections should be increased for this case, i.e.,.

(5) $$\begin{array}{c} \frac{\text{d}I}{\text{d}T}>0,\\ \text{or }N^{-1}\beta SI-\gamma I>0. \end{array}$$

Let us assume *S* = 1 in the beginning, then

(6) $$\begin{array}{c} \frac{\rho }{\sigma }=R_{0}>1. \end{array}$$

If *R*_0_ is more than 1, it is highly probable that an outbreak will occur in the future. On the other hand, *R*_0_ can be defined as a product of the contact rate *β* and infection time. *R*_0_ is the most effective parameter for choosing interventions. These three parameters can be decreased individually by vaccines, isolation, and antibiotics. *R*_0_ also provides five indications concerning an outbreak: whether or not it will turn out to be a pandemic; the initial increase rate of the epidemic; the final fraction size of the susceptible population that will get infected; the equilibrium fraction of susceptible individuals in a population; and the critical vaccination threshold.

### 2.5. Hyperparameter Optimization of the SIR Model

To achieve an optimized contact rate (*β*) and recovery  + mortality rate (*γ*), we referred to the Table 3 data and used the Optuna package with Python, hyperparameter optimization package. The resulting optimization parameters can be seen in Table 5. The root mean squared logarithmic error (RMSLE) score gives us the precise observed and estimated values of the optimization parameters. If the RMSLE value is high, the estimation is highly accurate. In this type of model, the main challenge is to estimate the best parameters to fit in the model. And if the model is based on time, the differential equations are necessary for modeling the real behaviors. Many parameter estimation methods are used in computational biology. The most common are Gauss Newton, Simulated annealing, Genetic algorithms, and so on. The main aim is to fit the model with the low costs and errors.

### 2.6. *S*-*R* Trend Analysis

**Figure 3:**
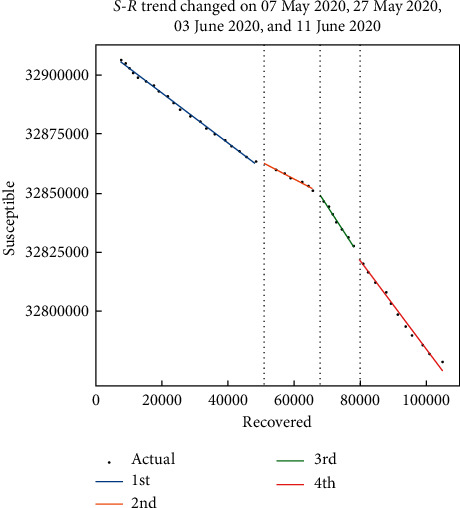
*S*-*R* trend analysis.

We can get some idea of the ending time of the epidemic using susceptible (*S*)–recovered (*R*) analysis. An epidemic is said to stop not only when everyone has been infected but also when nobody is freshly infected. *R*-*S* phase planes prove that a decrease in the number of susceptible persons is exponentially related to recoveries. This means some groups of the population will always avoid infection [40]. In Figure 3, the regression analysis is fitted to real data.

### 2.7. SIR-F Model

If we consider some cases of those who died before going to the hospital (confirmed), then the model is expanded:

(7) $$\begin{array}{c} \text{Confirmed}=I+R+F, \end{array}$$

where *R* = recovered and *F* = deaths.

Therefore, the model can be written as

(8) $$\begin{array}{c} S\overset{\beta I}{⟶}S^{*}\overset{\alpha_{1}}{⟶}F,\\ S^{*}\overset{1-\alpha_{1}}{⟶}I\overset{\gamma }{⟶}R,\\ I\overset{\alpha_{2}}{⟶}F, \end{array}$$

where *S*^*∗*^ is confirmed and uncategorized; *α*_1_ is the mortality rate of *S*^*∗*^ cases; *α*_2_ is the mortality rate of *I* cases (1/min); *β* is the effective contact rate (1/min); and *γ* is the recovery rate (1/min).

#### 2.7.1. Ordinary Differential Equations

(9) $$\begin{array}{c} \frac{\text{d}S}{\text{d}T}=-N^{-1}\beta S,\\ \frac{\text{d}I}{\text{d}T}=-N^{-1}\left(1-\alpha_{1}\right)\beta SI-\left(\gamma +\alpha_{2}\right)I,\\ \frac{\text{d}R}{\text{d}T}=\gamma I,\\ \frac{\text{d}F}{\text{d}T}=N^{-1}\alpha_{1}\beta SI+\alpha_{2}I, \end{array}$$

where *N* = *S* + *I* + *R* + *F* is the total population and *T* is the elapsed time from the start date.

(10) $$\begin{array}{c} \frac{\text{d}x}{\text{d}t}=-\rho xy,\\ \frac{\text{d}y}{\text{d}t}=\rho \left(1-\theta \right)xy-\left(\sigma +\kappa \right)y,\\ \frac{\text{d}z}{\text{d}t}=\sigma y,\\ \frac{\text{d}w}{\text{d}t}=\rho \theta xy+\kappa y,\\ R_{0}=\rho \left(1-\theta \right){\left(\sigma +\kappa \right)}^{-1}=\beta \left(1-\alpha_{1}\right){\left(\gamma +\alpha_{2}\right)}^{-1}. \end{array}$$

### 2.8. Hyperparameter Optimization of the SIR-F Model

Table 6 shows the hyperparameter optimization of the SIR-F Model.

### 2.9. Predicting the Future with SIR-F

Prediction of the future with the final parameters of the SIR-F model is also possible with the CovsirPhy: COVID-19 data with SIR model Python package in that it also considers *S*-*R* trend analysis.

#### 2.9.1. Worst-Case Scenario

**Figure 4:**
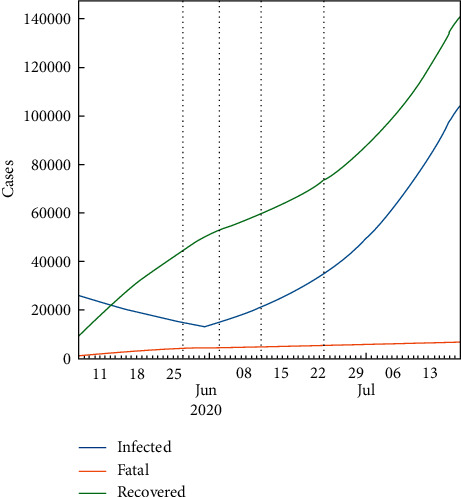
Predicted number of cases for the worst-case scenario using the SIR-F model for 30 days.

**Figure 5:**
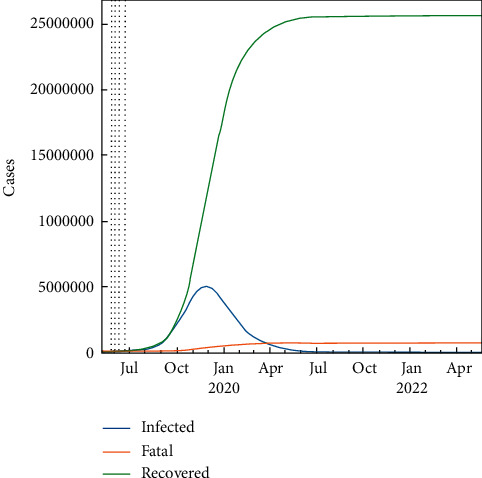
Predicted number of cases for the worst-case scenario using the SIR-F model for 700 days.

In the worst-case scenario, it is assumed that there is no lockdown, medicines, or vaccine for the population. This fundamental case is taken under consideration for the sake of comparison with the lockdown, medicine, and vaccine cases. As a result, the total number of infected people will be the highest in this case. So, if none of the interventions is invalid, the pure SIR-F model can predict the future within 30 days. This condition can accept the natural spread of the pandemic without any retarding effect on it. The prediction results are set out in Figure 4, from 22 June to display the infected, fatal, and recovered cases. Apparently, the spread will speed up in the near future such that both the infected and the recovered numbers will go up fast, but the fatality rate will continue to still remain less. Similarly, the prediction results are presented for 700 days in Figure 5, which shows that the epidemic will stop in the mid of October 2021.

**Figure 6:**
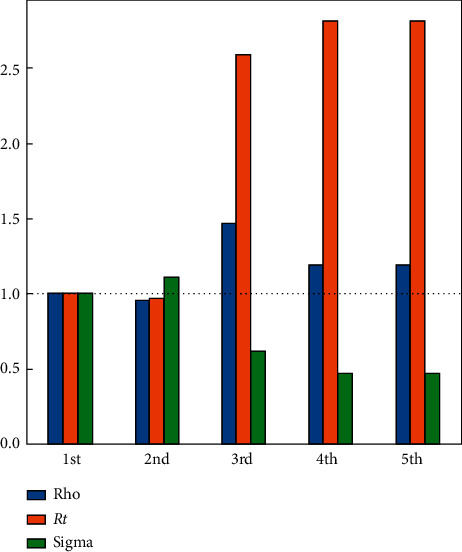
Ratio to the 1st phase parameters for the worst case.

The daily amount of fatal, infected, recovered, and susceptible numbers is listed in Table 7 where about 5 M people are infected with COVID-19, 25 M people have recovered, and there are 700 fatalities. Besides that, the pandemic seems to lose its effect completely after July 2021. Rho, sigma, and *R*_*t*_ (*R*_0_) parameters are shown in Figure 6 for the worst-case scenario using the SIR-F model. *R*_*t*_ (*R*_0_) parameter is explained in equations (4) and (5). The rho parameter shows that transitions to the infected state decreases after the 3rd stage and the reproductive rate increases at the 4th stage. The 5th stage is for forecasting the epidemic.

#### 2.9.2. Adding Interventions to the Models

In light of the rising numbers of cases and deaths, most governments have introduced interventions to reduce the spread of the virus causing COVID-19. In Europe and elsewhere, they have or are implementing measures to control the pandemic. These nonpharmaceutical interventions differ but generally incorporate social distancing (e.g., prohibiting large-scale gatherings and encouraging people not to associate outside their family units), fringe terminations, school terminations, and measures to seclude indicative people and their contacts [41].

### 2.10. Improved SIR-F Model with Closures and Lockdown

Some control factors should be added to the SIR-F model to investigate the effects of interventions such as school or market closures and lockdowns. In the case of lockdowns, the *gs* parameter defines the number of days that susceptible persons go out. Each age group in a population will go out for a different number of days. In Table 1, the population pyramid is defined for KSA, and we will estimate the average number of days that each the age group goes out. To precisely estimate the *gs* value, we will use the data listed in Table 2.

It is assumed that all schools and offices are closed, and that fifty per cent of people work remotely, and people are going out only on one day per week. Before the lockdown, the *gs* value was 6.07. After the start of the lockdown, the spending day's data have changed, as shown in Table 8. In Table 8, ten types of people with different age groups are listed with regard to who are going out during the pandemic. Ultimately, the new value of *gs* is 3.66. The number of people going out before the lockdown was necessary to estimate *gs* after the lockdown. We updated the population pyramid in Table 8 by updating the school, office, and other columns with the new *gs* value.

The new *gs* value is applied to the go-out table. We also assumed that workers go to their office one day a week, but they also go out for shopping and other emergency activities. This number is considered as 1 or zero due to inculturation of a more rule-based life here. The updated data are presented in Table 9, which will be used to implement the SIR-F model.

**Figure 7:**
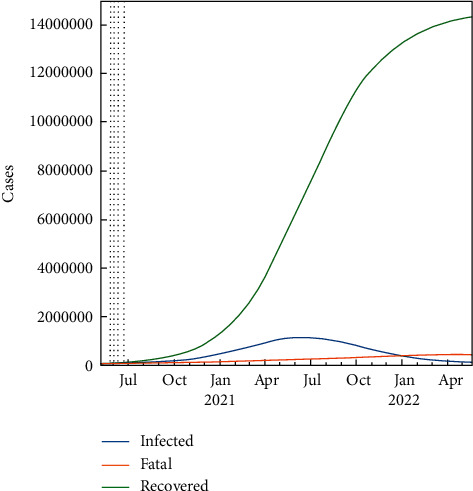
Predicted number of cases with lockdown using the SIR-F model for 700 days.

In the SIR-F model, gs is used as a control parameter of *β*. So, the *β* factor should change by the same amount as the change in *gs*. The effect of closedown begins at the start date of the third phase. The effect of lockdown can be seen by controlling the rho parameter in Figure 7, which will be explained in the next section.

### 2.11. Predict the Future with Lockdown and the Availability of New Medicines

#### 2.11.1. Lockdown

**Figure 8:**
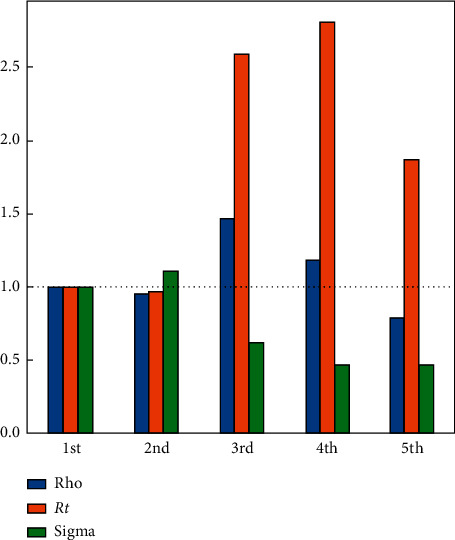
Ratio to 1st phase parameters for the lockdown case.

All the schools and offices have been closed since 26 July 2020. This lockdown precaution to counter the pandemic will not only decrease the maximum value of the effected persons but will also cause a delay of the infected bell curve to the late dates; ultimately, the pandemic period will be extended. Figure 7 verifies the prediction by decreasing and delaying of the infected curve to the April 2021. In Figure 7, the prediction results of three cases are shown for the next 700 days. Also, the pandemic period ends in April 2022. In Table 10, three cases have been given for the lockdown scenario; according to it, the number of infected persons is less than 1.5 M, the total number of recovered patients is about 14 M, and the total fatality rate decreased about four-hundred thousand people to about 392000. The ρ (rho) parameter of the 5th period is also decreased accordingly. In Figure 8, the transition parameter from S to I, which is denoted as *ρ* (rho), could not decrease drastically, which means that the infection rate is still keeping high, and we are far from the end of the epidemic. The most critical outcome of *ρ* is checking the interventions of the governments. Also, in Figure 8, the rho parameters are given for scenario 2, and it is clearly seen that school closure and lockdowns are not enough for stopping the epidemic in KSA.

#### 2.11.2. Effect of the Expected New Medicines

**Figure 9:**
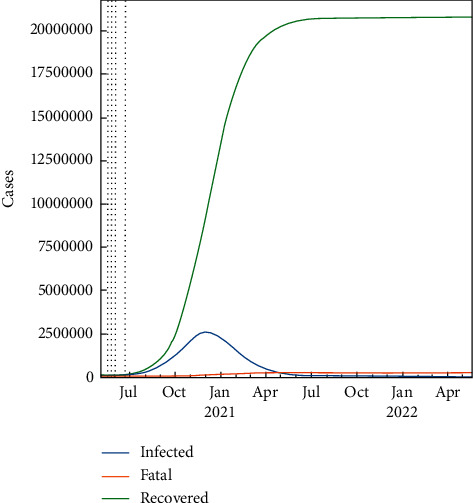
Predicted number of cases with medicine using the SIR-F model for 700 days.

**Figure 10:**
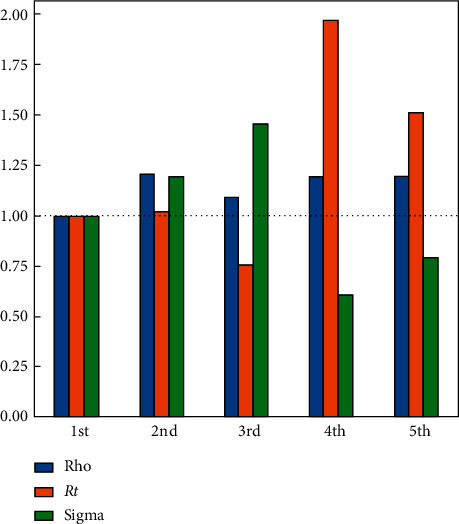
Ratio to 1st phase parameters for the case of medicine.

New drugs are essential for patients to recover rapidly from the disease. Medication repositioning methodology (i.e., finding successful competitors from the library of existing medications for various illnesses) is utilized to build up the medication possibilities to treat COVID-19. The new *α* and *γ* parameters are as in equation (11).  Figure 9 shows the predicted number of cases with medicine using the SIR-F model for 700 days, which mainly reduces the number of fatalities to 52000. Rho, sigma, and *R*_*t*_ (*R*_0_) parameters are shown in Figure 10 for the case with medicine using the SIR-F model, which indicates that the spread of infection is still very high, but the fatality rate is reduced due to medicine. The predicted number of SIR-F with medicine is shown in Table 11. This table shows around 2.5 M infected cases, which results in the shortening of the length of the pandemic bell curve.

(11) $$\begin{array}{c} \gamma_{\text{med}}=\frac{\tau \times \left(\text{Percentage of discharge}\right)}{\text{Total number of minutes with observation}}. \end{array}$$

## 3. Conclusions

The primary aim of this study was to evaluate the models for providing smart healthcare that are able to predict the onset of pandemics like COVID-19. This study proposes and implements SIR and SIR-F models with ML algorithms and presents both mathematical and numerical analyses and simulation results. The SIR epidemiological model is one of the oldest and has the most significant consequences of biological science. It contains the most significant highlights of the study of the virus disease transmission to be specific “Susceptible,” “Infected,” and “Recovered” people. The SIR model is applied to predict the data. Based on the SIR model, the pandemic will most probably be controlled by June 2021. The hyperparameters are presented in the various tables, and RMLSE is the primary accuracy metric to define the prediction error of real data and the SIR models. The reproductive rate was the most significant parameter for forecasting whether there will be a pandemic. Figures 4 and 6 show that despite the reproductive rate being low, the pandemic will increase, and the trend lines explain that the interventions by governments or individual isolation are not enough to stop the pandemic. Our proposed model proved to be successful in predicting peaks and the sizes of the COVID-19 outbreaks. Until the end of June 2021, the strategy of early detection and strict monitoring must continue to apply. With clear signs of an epidemic, individuals should be made aware of self-protection measures including frequent hand washing, either keeping soap on the hands for at least 20 seconds or using a hand sanitizer containing 60% alcohol, avoiding direct contact with sick people, keeping a distance of at least 6 feet from others, covering nose and mouth with a mask, using (and properly disposing of) a tissue to cover sneezes or coughs, and cleaning and sanitizing regularly touched items and surfaces every day. Although the government in a few places has slowly withdrawn lockdown restrictions, there is still a high possibility of outbreaks. The number of new cases of infection is increasing, and people should not let down their guard against this highly contagious disease.

## Figures and Tables

**Table 1:** Population pyramid of KSA [38].

Sl. No	Age	F	M	Year
0	0–4	1473864	1522376	2019
1	5–9	1446736	1494308	2019
2	10–14	1269502	1312481	2019
3	15–19	1092687	1142802	2019
4	20–24	1144722	1278548	2019

**Table 2:** Weekly time spending by people of various age groups.

#	Age first	Age last	Period of life	School days	Office days	Other days	Global	Philippines	KSA	Sweden
0	0	2	Nursery	3	0	0	0.052210	0.059807	0.052465	0.035809
1	3	5	Nursery school	4	0	1	0.051864	0.060893	0.052143	0.035727
2	6	10	Elementary school	5	0	1	0.084689	0.104088	0.083736	0.058956
3	11	13	Middle school	5	0	1	0.049386	0.060010	0.045212	0.034614
4	14	18	High school	6	0	1	0.079324	0.097064	0.067263	0.053759
5	19	25	University/work	3	3	1	0.107659	0.129404	0.101481	0.081871
6	26	35	Work	0	6	1	0.152774	0.156425	0.189787	0.138543
7	36	45	Work	0	5	1	0.131630	0.122671	0.199444	0.123699
8	46	55	Work	0	5	1	0.116396	0.095804	0.122208	0.132422
9	56	65	Work	0	5	1	0.088096	0.065197	0.055298	0.115113
10	66	75	Retired	0	0	4	0.055083	0.033720	0.021573	0.107004
11	76	85	Retired	0	0	3	0.024309	0.012498	0.007854	0.061383
12	86	95	Retired	0	0	2	0.006579	0.002420	0.001536	0.021099

**Table 3:** Number of cases.

Date	Confirmed	Infected	Fatal	Recovered
2020-06-06	98869	26402	676	71791
2020-06-07	101914	28385	712	72817
2020-06-08	105283	30013	746	74524
2020-06-09	108571	31449	783	76339
2020-06-10	112288	33515	819	77954

**Table 4:** Statistical summary of EDA.

	Confirmed	Infected	Fatal	Recovered
Count	93	93	93	93
Mean	31373.5	14129.3	202.1	17042.2
Std	34579.8	11834.1	214.5	24913.9
Min	20	19	0	1
25%	1885	1536	21	328
50%	16299	13948	136	2215
75%	57345	26402	320	28748
Max	112288	33515	819	77954

**Table 5:** Hyperparameter estimation of the basic SIR model.

	Start	End	Population	ODE	Rho	Sigma	tau min	*R*_0_	1/beta (day)	1/gamma (day)	RMSLE	Trials
1st	10 May 2020	18 May 2020	32,94 M	SIR	0.07831	0.07383	1440	1.06	12	13	0.02243	85
2nd	19 May 2020	25 May 2020	32,94 M	SIR	0.08977	0.08436	1440	1.06	11	11	0.00902	84
3rd	26 May 2020	02 Jun 2020	32,94 M	SIR	0.06981	0.10682	1440	0.65	14	9	0.01957	85
4th	03 Jun 2020	10 Jun 2020	32,94 M	SIR	0.10942	0.05181	1440	2.11	9	19	0.00816	85

**Table 6:** Hyperparameter estimation of SIR-F model.

Sl. No	Start	End	ODE	Rho	Sigma	*R*_*t*_	1/beta (day)	1/gamma (day)	RMSLE	Trials	Theta	Kappa	Alpha1	1/alpha2 (day)
1st	06 May 2020	12 May 2020	SIR-F	0.06916	0.04864	1.41	14	20	0.02435	234	0.00089	0.00036	0.001	2748
2nd	13 May 2020	23 May 2020	SIR-F	0.08907	0.06946	1.28	11	14	0.06279	164	0.00186	0.00018	0.002	5444
3rd	24 May 2020	30 May 2020	SIR-F	0.06831	0.10052	0.67	14	9	0.03131	158	0.00113	0.00065	0.001	1529
4th	31 May 2020	10 Jun 2020	SIR-F	0.09483	0.05649	1.65	10	17	0.08880	162	0.01801	0.00002	0.018	47464

**Table 7:** Predicted number of SIR-F without lockdown.

Sl. No.	Date	Fatal	Infected	Recovered	Susceptible
737	14 May 2022	700004	16	25603793	6636187
738	15 May 2022	700004	16	25603794	6636187
739	16 May 2022	700004	15	25603795	6636187
740	17 May 2022	700004	15	25603795	6636186
741	18 May 2022	700004	15	25603796	6636186
742	19 May 2022	700004	14	25603797	6636186
743	20 May 2022	700004	14	25603797	6636186

**Table 8:** Spending days after lockdown.

Sl No	Age_first	Age_last	Period_of_life	School	Office	Others	Portion
0	0	2	Nursery	0	0.0	1	0.052465
1	3	5	Nursery school	0	0.0	2	0.052143
2	6	10	Elementary school	0	0.0	2	0.083736
3	11	13	Middle school	0	0.0	2	0.045212
4	14	18	High school	0	0.0	2	0.067263
5	19	25	University/work	0	1.5	2	0.101481
6	26	35	Work	0	3.0	2	0.189787
7	36	45	Work	0	2.5	2	0.199444
8	46	55	Work	0	2.5	2	0.122208
9	56	65	Work	0	2.5	2	0.055298
10	66	75	Retired	0	0.0	4	0.021573
11	76	85	Retired	0	0.0	3	0.007854
12	86	95	Retired	0	0.0	2	0.001536

**Table 9:** Spending days updated with the new *gs* after lockdown.

Sl No	Age_first	Age_last	Period_of_life	School	Office	Others	Portion
0	0	2	Nursery	0	0.0	3.0	0.052465
1	3	5	Nursery school	0	0.0	3.0	0.052143
2	6	10	Elementary school	0	0.0	3.0	0.083736
3	11	13	Middle school	0	0.0	3.0	0.045212
4	14	18	High school	0	0.0	3.0	0.067263
5	19	25	University/work	0	1.0	3.0	0.101481
6	26	35	Work	0	1.0	3.0	0.189787
7	36	45	Work	0	1.0	3.0	0.199444
8	46	55	Work	0	1.0	3.0	0.122208
9	56	65	Work	0	1.0	3.0	0.055298
10	66	75	Retired	0	0.0	3.0	0.021573
11	76	85	Retired	0	0.0	3.0	0.007854
12	86	95	Retired	0	0.0	3.0	0.001536

**Table 10:** Predicted number of the SIR-F with lockdown.

	Date	Confirmed	Fatal	Infected	Recovered
737	14 May 2022	391607	78750	14280299	18189343
738	15 May 2022	391696	77859	14283571	18186873
739	16 May 2022	391784	76978	14286807	18184431
740	17 May 2022	391871	76106	14290005	18182017
741	18 May 2022	391957	75244	14293168	18179631
742	19 May 2022	392042	74391	14296294	18177272
743	20 May 2022	392087	73940	14297949	18176024

**Table 11:** Predicted number of SIR-F with medicine.

	Date	Confirmed	Fatal	Infected	Recovered
737	14 May 2022	52082	87	20784310	12103520
738	15 May 2022	52082	85	20784314	12103517
739	16 May 2022	52082	83	20784319	12103515
740	17 May 2022	52082	81	20784324	12103512
741	18 May 2022	52082	80	20784328	12103510
742	19 May 2022	52082	78	20784332	12103507
743	20 May 2022	52082	77	20784335	12103506

## Data Availability

Publicly available datasets were analyzed in this study. These datasets can be found at https://github.com/CSSEGISandData/COVID-19.
